# PReS-FINAL-2261: Prevalence of orofacial symptoms and signs in patients with juvenile fibromyalgia

**DOI:** 10.1186/1546-0096-11-S2-P251

**Published:** 2013-12-05

**Authors:** C Len, L Zwir, M Fraga, MT Terreri

**Affiliations:** 1 Pediatrics, UNIFESP, Sao Paulo, Brazil

## Introduction

Fibromyalgia may coexist with other clinical conditions such temporomandibular disorders (TMD). Temporomandibular disorder is a term embracing clinical disorders that involve the masticatory musculature, the temporomandibular joints, and associated structures.

## Objectives

The purpose of this study was to assess the prevalence of orofacial symptoms and signs in patients with juvenile fibromyalgia.

## Methods

Twenty-eight consecutive patients (22 girls) who presented to our outpatient pediatric rheumatology clinic and fulfilled the ACR criteria of fibromyalgia were included in this study. All patients underwent a rheumatologic examination performed by a pediatric rheumatologist, and an orofacial examination performed by a single dentist at the same data. The patients were interviewed according to a standardized questionnaire concerning the presence of orofacial pain and functional impairment and were submitted to a clinical evaluation following a structured protocol.

## Results

The mean age at the evaluation was 13 years (range 8- 18 years) and the mean follow-up time was 3.3 years (0.3 to 12). Orofacial symptoms were reported in 14 (50%) of patients. The most common subjective symptoms were impaired ability with chewing (50%), pain in function (28.6%), and pain at rest (17.8%). We found that the vast majority of our patients (89.3%)reported pain on palpation in at least two of six sites in the orofacial region, but only 7 (25%) had pain during mandibular movements, and nobody had restrictions to open their mouths 40 mm or more.

## Conclusion

Although palpation of the orofacial region is not included in the diagnostic criteria for fibromyalgia, a large number of juvenile patients presented with pain on palpation in this region. This study suggests the need for interdisciplinary strategies to effectively diagnose and treat this chronic condition.

## Disclosure of interest

None declared.

